# Clinical characteristics and survival in patients with heart failure experiencing in hospital cardiac arrest

**DOI:** 10.1038/s41598-022-09510-4

**Published:** 2022-04-05

**Authors:** Emma Aune, John McMurray, Peter Lundgren, Naveed Sattar, Johan Israelsson, Per Nordberg, Johan Herlitz, Araz Rawshani

**Affiliations:** 1 Institute of Medicine, University of Gothenburg, Gothenburg, Sweden; 2 British Heart Foundation (BHF) Cardiovascular Research Centre, University of Glasgow, Glasgow, UK; 3 Prehospen-Centre for Prehospital Research, University of Borås, Borås, Sweden; 4 Department of Internal Medicine, Division of Cardiology, Kalmar County Hospital, Region Kalmar County, Sweden; 5 Faculty of Health and Life Sciences, Linnaeus University, Kalmar, Sweden; 6 Department of Medicine, Center for Resuscitation Science, Karolinska Institute, Solna, Sweden; 7 The Swedish Registry of Cardiopulmonary Resuscitation, Gothenburg, Sweden

**Keywords:** Cardiology, Medical research

## Abstract

In patients with heart failure (HF) who suffered in-hospital cardiac arrest (IHCA), little is known about the characteristics, survival and neurological outcome. We used the Swedish Registry of Cardiopulmonary Resuscitation to study this, including patients aged ≥ 18 years suffering IHCA (2008–2019), categorised as HF alone, HF with acute myocardial infarction (AMI), AMI alone, or other. Odds ratios (OR) for 30-day survival, trends in 30-day survival, and the implication of HF phenotype was studied. 6378 patients had HF alone, 2111 had HF with AMI, 4210 had AMI alone. Crude 5-year survival was 9.6% for HF alone, 12.9% for HF with AMI and 34.6% for AMI alone. The 5-year survival was 7.9% for patients with HF and left ventricular ejection fraction (LVEF) ≥ 50%, 15.4% for LVEF < 40% and 12.3% for LVEF 40–49%. Compared with AMI alone, adjusted OR (95% CI) for 30-day survival was 0.66 (0.60–0.74) for HF alone, and 0.49 (0.43–0.57) for HF with AMI. OR for 30-day survival in 2017–2019 compared with 2008–2010 were 1.55 (1.24–1.93) for AMI alone, 1.37 (1.00–1.87) for HF with AMI and 1.30 (1.07–1.58) for HF alone. Survivors with HF had good neurological outcome in 92% of cases.

## Introduction

Individuals with heart failure (HF) are at higher risk of cardiac arrest than people in the general population, and even compared to those with other types of cardiovascular disease. Around half of all deaths in patients with HF occur suddenly^1–3^. Many or even most of these deaths in patients with HF and reduced ejection fraction (HFrEF) are thought to be due to ventricular arrhythmias, confirmed by the effectiveness of implanted defibrillating devices (relative risk typically reduced by 20–50%), although non-cardiac events such as aortic rupture and stroke may also result in rapid death^4–14^. Less is known about the risk and causes of cardiac arrest in patients with HF and preserved ejection fraction (HFpEF). In addition, little is known about the incidence and outcome of cardiac arrest in either major HF phenotype or the more recently defined HF with mid-range ejection fraction (HFmrEF)^1,15^.

The Swedish Registry of Cardiopulmonary Resuscitation (SRCR) is a nationwide registry recording cardiac arrests that prompt resuscitation attempts in all hospitals in Sweden^16,17^. We aimed to study in-hospital cardiac arrests in patients with HF, describe their characteristics anddetermine short and long term survival, neurological outcome, and trends in survival during 2008 to 2019.

## Methods

### The Swedish registry of cardiopulmonary resuscitation

The vast majority of in-hospital cardiac arrests (IHCA) in Sweden are registered in the SRCR. The registry was launched in 1990, recording out-of-hospital cardiac arrests (OHCA) and has since 2005 also recorded IHCA. The coverage of the SRCR has been high since 2008, and from December 2019 all 74 qualifying hospitals in Sweden report to SRCR. A hospital was defined as a care facility which has its own cardiac arrest response team and intensive care unit with the possibility of providing post-resuscitation care. More details of the registry has been described previously^16–18^.

The SRCR was designed to comply with the Utstein style of reporting IHCA^19^. Events are registered in three protocols; the first includes data regarding the cardiac arrest event and treatments provided by hospital staff adjacent to the event. The second protocol includes follow-up data from medical records on e.g. comorbidity, etiology, post-resuscitation care, survival and neurological function. The third protocol is based on patient reported data collected during a telephone follow-up at 3–6 months after IHCA.

The definition of IHCA used for the registry was an unresponsive patient with apnea or abnormal breathing (agonal or gasping respiration), where cardiopulmonary resuscitation (CPR) and/or defibrillations was initiated within the walls of the hospital.

HF is in the SRCR defined as a diagnosis of HF made by the clinician treating the patient, previous to the IHCA, during the same admission or during a previous admission. Similarly, acute myocardial infarction (MI) was defined as onset of MI within 72 h prior to IHCA, diagnosed by the treating clinician of the patient. Comorbidities in the SCRC (i.e. cancer, diabetes, HF, MI and stroke) are likewise defined as a clinical diagnosis. Initial (*i.e.* first recorded) rhythm is categorized as either shockable (ventricular fibrillation [VF], pulseless ventricular tachycardia [pVT]) or non-shockable (pulseless electrical activity [PEA] or asystole), based on the first recorded electrocardiogram (ECG) and, secondarily, the recommendation produced by the connected defibrillator.

Neurological function is assessed using the cerebral performance category (CPC) scale, which ranges from 1 to 5 ((1) good cerebral performance; (2) moderate cerebral disability; (3) severe cerebral disability; (4) coma or vegetative state; (5) brain death). CPC 1–2 is normally considered a good neurological outcome post cardiac arrest^20^. CPC is assessed at discharge among patients discharged alive.

### Study population

The investigation was a retrospective inception cohort design of persons with IHCA in the SRCR between 2008-01-01 and 2019-12-31.

Patients aged 18 years or older were included in the study which covered the period 2008-01-01 to 2019-12-31. The patients were grouped into the following four categories based on their previous diagnoses: HF alone (i.e. patients with prevalent HF without acute MI who suffered an IHCA), HF with acute MI (i.e. patients with prevalent HF and an acute MI who suffered an IHCA), acute MI alone (i.e. patients with acute MI without HF who suffered an IHCA), and other (i.e. patients who suffered an IHCA not included in previously mentioned categories; no HF, no acute MI). HF was the exposure of main interest, whereas acute MI served as the comparator. We chose the acute MI alone group as the comparator as acute MI is the most common cause of IHCA in the SRCR, and it is a well-studied and defined group^21,22^.

All cases of HF reported in the SRCR were included in our analyses, irrespective of etiology, duration, left ventricular ejection fraction (LVEF) and New York Heart Association (NYHA) functional class. The entire medical record was examined for information on a diagnosis of HF prior to IHCA. For the subgroup analysis of LVEF, patients with HF without an acute MI, who had a recorded LVEF measured within 6 months preceding the IHCA were categorised as follows: LVEF < 40% (HFrEF), 40–49% (HFmrEF) and ≥ 50% (HFpEF). Patients with HF, without an acute MI, who did not have a recent measurement of LVEF were categorized separately as a fourth group. As a sensitivity analysis, we also stratified patients with HF, without acute MI, into just two groups based on LVEF; less than 50% or 50% or more.

Both monitoring and a shockable initial rhythm is well established favourable prognostic factors for survival, thus, as additional sensitivity analysis, we separately stratified our patients upon whether monitoring was performed or not at the time of IHCA, and whether the initial rhythm was shockable or not.

### Statistical methods

Baseline characteristics are presented in means and medians, with appropriate measures of dispersions. The Kaplan–Meier method was used to describe survival distributions after IHCA. Logistic regression was used to calculate probabilities for 30-days survival. From this, we extracted survival percentages at 1 and 5 years. We performed subgroup analyses according to sex, age (grouped by 18–49 years, 50–69 years and 70 years or older) and diabetes status. Covariate adjustment was made for age, sex, time from arrest to cardiopulmonary resuscitation (CPR), initial rhythm and witnessed status among these subgroups.

We also used logistic regression to compute trends in 30-day survival, during 2008 to 2019; these models were adjusted for age and sex.

Furthermore, logistic regression was utilised to visualize the association between LVEF and 30-days survival. This was done by expanding LVEF into a restricted cubic spline with 4 knots, while adjusting for age, sex, time from arrest to CPR, initial rhythm and witnessed status.

All analyses were performed using R (version 4.0.3). The study has been approved by the Swedish Ethical Review Authority, in accordance with the Helsinki Declaration, and informed consent was provided upon enrollment in the registry.

## Results

A total of 29,868 patients were enrolled in the SRCR during the study period, of whom 26,419 patients were at least 18 years of age. A total of 6378 patients (24%) had HF alone, 2111 (8%) had HF with an acute MI, 4210 (16%) had acute MI alone and 13,720 (52%) consisted of other patients, with no previous HF and no acute MI. Overall, mean age was 72.5 years (standard deviation [SD] 13.5 years) and 38.4% were women.

### Baseline characteristics

#### Baseline characteristics of patients with HF, acute MI or both

Patients with HF, with or without acute MI, were older than patients with acute MI alone or other (mean age HF alone 75.6 years, HF with acute MI 75.7 years, acute MI alone 72.3 years and other 70.6 years; *p* < 0.001). The proportion of women was similar in the groups of patients with HF and/or acute MI, at around 35% and slightly higher among others at 42%. The prevalence of diabetes was considerably higher in patients with HF compared to those with acute MI alone or other (HF alone 36.1%, HF with acute MI 38.4%, acute MI alone 22.9% and other 21.2%; *p* < 0.001).

#### Baseline characteristics of patients with HF alone according to LVEF

In patients with HF alone, with a LVEF measurement available (55% of patients in this group), those in the lowest LVEF category were youngest, and those in the highest LVEF category the oldest (LVEF < 40% mean age 71.8 years, LVEF 40–49% 75.4 years and LVEF ≥ 50% 76.0 years; *p* < 0.001). Patients without a LVEF measurement were older still (mean age 78.2 years), and 40% were women. The proportions of women also increased across these categories, from 26.1% among those with a LVEF < 40%, through 29.0% in patients with LVEF 40–49% to 49.5% in those with LVEF ≥ 50% (*p* < 0.001). History of MI (prior MI) varied substantially across these 4 groups—49.5%, 38.0%, 28.9% and 32.1%, respectively (*p* < 0.001)—whereas the prevalence of diabetes did not.

#### Monitoring and initial rhythm in patients with HF, acute MI or both

The proportion of patients having ECG monitoring at the time of IHCA was much lower (47.5%) in patients with HF alone and in other (44.7%) than in patients with acute MI with (71.5%) or without HF (78.1%); *p* < 0.001. There was a notable difference in the proportion of patients with an initial shockable rhythm, which was identified in only 26.5% of patients with HF alone but in 41.2% of HF patients with an acute MI, in 49.4% of patients with an acute MI alone. Among other, only 16.4% had an initial shockable rhythm (*p* < 0.001) (Table 1). Asystole was the first recorded rhythm in 40.5% of patients with HF alone, 31.5% of patients with HF with acute MI and 25.5% of patients with acute MI alone (Supplementary Fig. 1A).

**Table 1:** Baseline characteristics of 26,419 cases of in-hospital cardiac arrest.

	All patients						Patients with HF alone					
	Other	HF alone	HF with acute MI	Acute MI alone	*p*	SMD	HF no LVEF measurement	LVEF < 40%	LVEF 40–49%	LVEF ≥ 50%	*p*	SMD
*n*	13,720	6378	2111	4210			2875	2032	774	697		
Age—mean (SD)	70.59 (14.60)	75.56 (11.39)	75.66 (10.37)	72.30 (11.94)	< 0.001	0.249	78.17 (10.56)	71.80 (12.15)	75.38 (10.60)	75.97 (10.17)	< 0.001	0.296
Woman—*n* (%)	5753 (41.9)	2248 (35.3)	664 (31.5)	1481 (35.2)	< 0.001	0.109	1149 (40.0)	530 (26.1)	224 (29.0)	345 (49.5)	< 0.001	0.286
LVEF (%)—mean (SD)	52.82 (9.95)	35.78 (13.28)	33.94 (11.39)	47.78 (11.44)	< 0.001	1.003	NA	26.51 (7.42)	42.47 (2.55)	55.35 (5.90)	< 0.001	NA
HF with LVEF < 50%—*n* (%)	NA	697 (19.9)	141 (11.2)	NA	NA	NA	NA	0 (0.0)	0 (0.0)	697 (100.0)	NA	NA
Comorbidities												
MI, previous—*n* (%)	1671 (12.7)	2306 (38.1)	1063 (53.1)	1005 (24.8)	< 0.001	0.512	864 (32.1)	964 (49.5)	284 (38.0)	194 ( 28.9)	< 0.001	0.235
Stroke, ongoing—*n* (%)	517 (4.0)	138 (2.2)	49 (2.4)	77 (1.9)	< 0.001	0.064	70 (2.6)	42 (2.1)	16 (2.1)	10 (1.5)	0.350	0.039
Stroke, previous—*n* (%)	1448 (10.9)	859 (13.6)	280 (13.4)	429 (10.4)	< 0.001	0.063	420 (14.9)	253 (12.5)	102 (13.3)	84 (12.1)	0.067	0.044
Cancer, any—*n* (%)	3011 (22.9)	1027 (16.5)	261 (12.6)	555 (13.5)	< 0.001	0.150	502 (18.0)	277 (13.9)	133 (17.4)	115 (16.9)	0.002	0.058
Diabetes—*n* (%)	2834 (21.2)	2287 (36.1)	805 (38.4)	949 (22.9)	< 0.001	0.240	956 (33.7)	770 (38.1)	286 (37.0)	275 (39.5)	0.003	0.064
Location of cardiac arrest												
CCU—*n* (%)	1345 (9.8)	1135 (17.8)	707 (33.5)	1101 (26.2)			332 (11.5)	560 (27.6)	140 (18.1)	103 (14.8)		
ICU—*n* (%)	1382 (10.1)	574 (9.0)	205 (9.7)	230 (5.5)			203 (7.1)	251 (12.4)	76 ( 9.8)	44 (6.3)		
OR—*n* (%)	385 (2.8)	96 (1.5)	13 (0.6)	13 (0.3)			50 (1.7)	24 (1.2)	11 (1.4)	11 (1.6)		
ER—*n* (%)	1524 (11.1)	520 (8.2)	181 (8.6)	564 (13.4)			275 (9.6)	138 (6.8)	57 (7.4)	50 (7.2)		
Outpatient lab, radiology—*n* (%)	680 (5.0)	231 (3.6)	52 (2.5)	106 (2.5)			96 (3.3)	65 (3.2)	34 (4.4)	36 (5.2)		
Cathlab—*n* (%)	418 (3.0)	117 (1.8)	249 (11.8)	1235 (29.3)			27 (0.9)	61 (3.0)	17 (2.2)	12 (1.7)		
IMW—*n* (%)	115 (0.8)	38 (0.6)	5 (0.2)	4 (0.1)			17 (0.6)	9 (0.4)	6 (0.8)	6 (0.9)		
Regular ward—*n* (%)	7518 (54.8)	3533 (55.4)	673 (31.9)	879 (20.9)			1813 (63.1)	875 (43.1)	422 (54.5)	423 (60.7)		
Other—*n* (%)	353 (2.6)	134 (2.1)	26 (1.2)	78 (1.9)			62 (2.2)	49 (2.4)	11 (1.4)	12 (1.7)		
Monitoring, witnessed status and initial rhythm												
ECG monitoring—*n* (%)	6035 (44.7)	2991 (47.5)	1497 (71.5)	3260 (78.1)	< 0.001	0.446	1063 (37.5)	1258 (62.6)	397 (51.6)	273 (39.7)	< 0.001	0.297
Witnessed arrest—*n* (%)	10,552 (78.3)	4865 (77.6)	1819 (87.4)	3791 (91.0)	< 0.001	0.229	2052 (72.8)	1678 (83.6)	608 (80.4)	527 (76.8)	< 0.001	0.148
Shockable initial rhythm—*n* (%)	2053 (16.4)	1576 (26.5)	822 (41.2)	1964 (49.4)	< 0.001	0.421	469 (17.6)	801 (42.1)	197 (27.2)	109 (16.8)	< 0.001	0.326
Time delays												
Time to alert—median (IQR)	1.00 [1.00, 1.00]	1.00 [1.00, 1.00]	1.00 [1.00, 1.00]	1.00 [1.00, 1.00]	< 0.001	0.021	1.00 [1.00, 1.00]	1.00 [1.00, 1.00]	1.00 [1.00, 1.00]	1.00 [1.00, 1.00]	0.013	0.029
Time to CPR—median (IQR)	0.00 [0.00, 1.00]	0.00 [0.00, 1.00]	0.00 [0.00, 1.00]	0.00 [0.00, 0.00]	< 0.001	0.026	0.00 [0.00, 1.00]	0.00 [0.00, 1.00]	0.00 [0.00, 1.00]	0.00 [0.00, 1.00]	0.005	0.038
Time to defibrillation—median (IQR)	3.00 [1.00, 7.00]	2.00 [1.00, 5.00]	2.00 [1.00, 4.00]	1.00 [0.00, 3.00]	< 0.001	0.053	3.00 [1.00, 7.00]	2.00 [1.00, 4.00]	2.00 [1.00, 7.00]	3.00 [2.00, 7.00]	< 0.001	0.052
Acute management												
CPR before rescue team arrival—*n* (%)	11,475 (92.4)	5411 (92.8)	1762 (92.2)	3256 (91.3)	0.067	0.029	2531 (93.2)	1617 (91.6)	653 (92.9)	610 (94.0)	0.129	0.048
Defibrillated before rescue team arrival—*n* (%)	1221 (12.7)	941 (20.1)	523 (32.4)	1239 (40.4)	< 0.001	0.376	299 (13.9)	449 (30.9)	121 (21.3)	72 (14.4)	< 0.001	0.238
Defibrillated, any—*n* (%)	3117 (23.2)	2090 (33.4)	989 (47.5)	2277 (54.7)	< 0.001	0.384	700 (24.9)	938 (46.9)	285 (37.4)	167 (24.5)	< 0.001	0.285
Ventilated—*n* (%)	9230 (81.2)	4509 (84.0)	1448 (82.6)	2453 (76.2)	< 0.001	0.104	2170 (86.2)	1311 (82.0)	528 (81.6)	500 (82.1)	< 0.001	0.063
Intubated—*n* (%)	7127 (53.9)	2935 (47.7)	938 (45.8)	1751 (42.9)	< 0.001	0.117	1383 (49.9)	838 (42.9)	366 (48.9)	348 (51.4)	< 0.001	0.089
Mechanical compressions—*n* (%)	1317 (10.2)	483 ( 8.0)	230 (11.4)	616 (15.3)	< 0.001	0.122	225 ( 8.3)	160 (8.3)	53 (7.2)	45 (6.8)	0.451	0.036
Adrenaline given—*n* (%)	9028 (67.8)	4132 (66.6)	1374 (66.6)	2333 (57.1)	< 0.001	0.111	1978 (70.5)	1182 (60.4)	507 (67.0)	465 (68.2)	< 0.001	0.111
Antiarrythmics given—*n* (%)	1350 (10.6)	977 (16.3)	517 (25.9)	991 (25.0)	< 0.001	0.238	314 (11.7)	467 (24.4)	120 (16.4)	76 (11.6)	< 0.001	0.193

CPR = cardiopulmonary resuscitation; ECG = electrocardiogram; HF = heart failure; IQR = interquartile range; LVEF = left ventricular ejection fraction; MI = myocardial infarction; SD = standard deviation; SMD = standard mean deviation.

#### Monitoring and initial rhythm in patients with HF alone according to LVEF

The proportion of patients undergoing ECG monitoring during the time of arrest was highest among patients with the lowest LVEF (62.6% among LVEF < 40%, 51.6% for LVEF 40–49%, 39.7% among LVEF ≥ 50%; *p* < 0.001). For HF patients without a recent LVEF measurement the percentage undergoing ECG monitoring at time of IHCA was the lowest of all (37.5%). The occurrence of a shockable initial rhythm was lowest among patients with a high LVEF or no recent measurement (16.8% for LVEF ≥ 50% and 17.6% for those without a recent measurement) compared to those with the lowest LVEF (42.1% for LVEF < 40%). Patients with a LVEF of 40–49% had a shockable initial rhythm in 27.2% of cases (Table 1). Asystole was noted as the first recorded rhythm in 28.8% for LVEF < 40%, 40.1% for LVEF 40–49% and 46.5% for LVEF ≥ 50% (Supplementary Fig. 1B).

### Clinical status after CPR, interventions and outcomes

#### Neurological outcome at discharge

**Figure 1:**
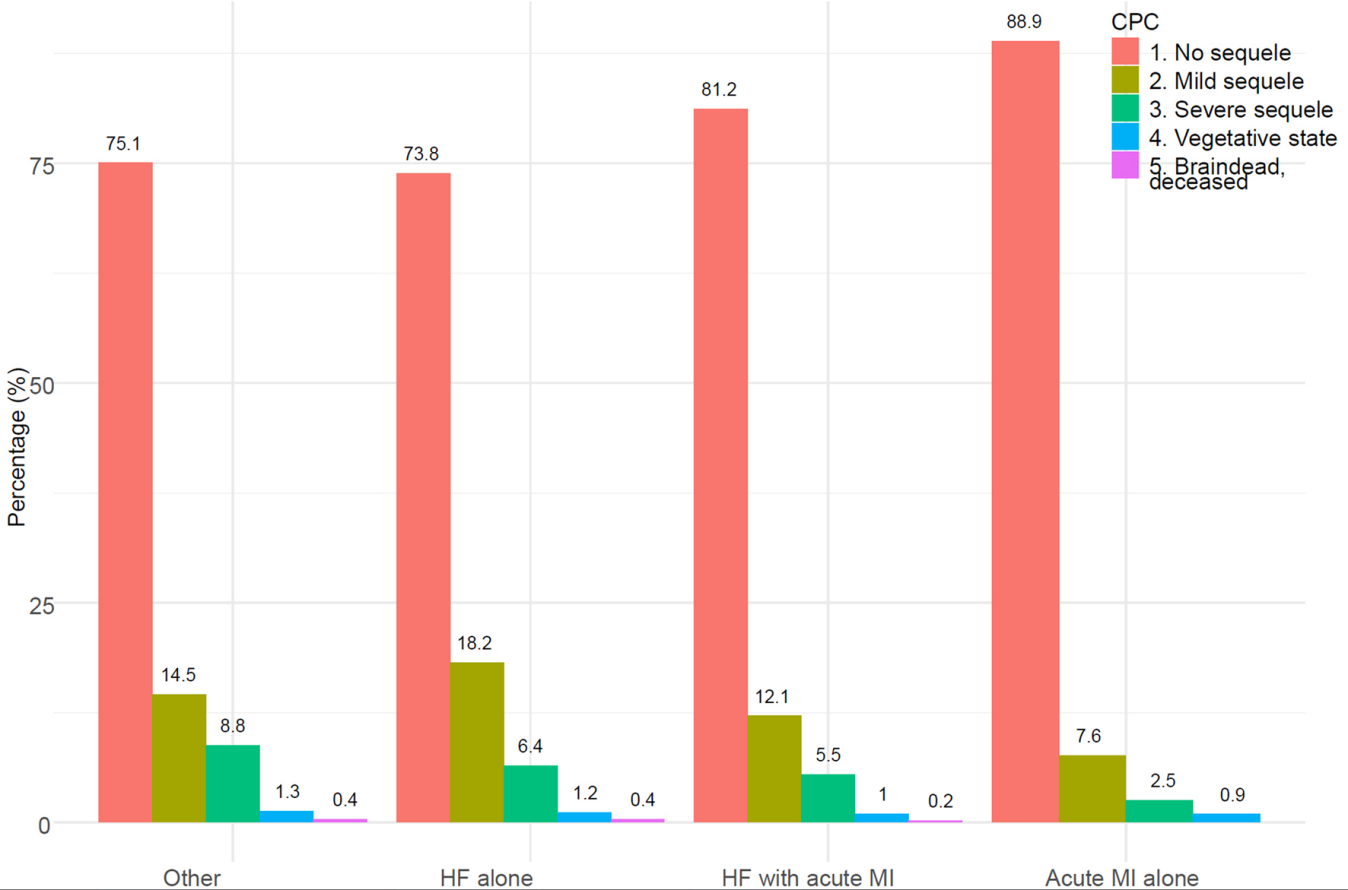
Neurological deficits measured as cerebral performance category in patients discharged alive. CPC scores among patients who were discharged alive. HF = heart failure; MI = myocardial infarction.

Figure 1 shows CPC scores for patients who were discharged alive. The vast majority, in all groups examined, scored CPC 1 or 2 (i.e. good neurological outcome). The proportion having CPC 1 or 2 was 92.0% in patients with HF alone, 93.3% in patients with HF and acute MI and 96.5% in cases with acute MI alone.

### Unadjusted 30-day survival

#### Comparison of patients with HF, acute MI or both

Survival at 30 days was lower in patients with HF alone (24.9%), or HF with acute MI (26.4%), compared with acute MI alone (44.8%); *p* < 0.001. Other patients had an unadjusted 30-day survival rate at 29.6% (Table 2).

**Table 2:** Status post CPR, interventions and outcome in 26,419 cases of in-hospital cardiac arrest.

	All patients						Patients with HF alone					
	Other	HF alone	HF with acute MI	Acute MI alone	*p*	SMD	HF no LVEF measurement	LVEF < 40%	LVEF 40–49%	LVEF ≥ 50%	*p*	SMD
Status post CPR												
Consciousness—*n* (%)	1708 (14.0)	717 (12.5)	333 (17.8)	872 (24.8)	< 0.001	0.178	218 (8.1)	332 (19.1)	99 (14.3)	68 (10.5)	< 0.001	0.182
Breathing—*n* (%)	2679 (22.1)	1169 (20.5)	513 (27.6)	1195 (34.4)	< 0.001	0.179	422 (15.9)	476 (27.5)	158 (23.1)	113 (17.7)	< 0.001	0.165
Pulse—*n* (%)	2736 (24.0)	1202 (22.4)	495 (27.9)	1116 (34.0)	< 0.001	0.144	447 (18.0)	484 (29.5)	163 (25.4)	108 (18.4)	< 0.001	0.165
Interventions												
Angiography—*n* (%)	219 (9.2)	115 (10.9)	118 (41.0)	509 (60.6)	< 0.001	0.745	19 (5.6)	66 (16.1)	24 (15.3)	6 ( 4.1)	< 0.001	0.256
PCI—*n* (%)	109 (4.6)	41 (3.9)	112 (38.9)	543 (64.6)	< 0.001	0.953	8 (2.4)	19 (4.6)	10 (6.4)	4 (2.7)	0.117	0.117
Pacemaker implanted—*n* (%)	191 (13.2)	96 (13.8)	26 (14.2)	46 (8.9)	0.045	0.086	18 (8.1)	53 (18.9)	13 (13.0)	12 (12.8)	0.006	0.162
ICD implanted—*n* (%)	39 (2.7)	60 (8.6)	11 (6.0)	21 (4.1)	< 0.001	0.146	6 (2.7)	39 (13.9)	10 (10.0)	5 (5.3)	< 0.001	0.241
Outcome												
Survival at 30 days—*n* (%)	4057 (29.6)	1590 (24.9)	558 (26.4)	1887 (44.8)	< 0.001	0.224	478 (16.6)	716 (35.2)	235 (30.4)	161 (23.1)	< 0.001	0.244

CPR = cardiopulmonary resuscitation; HF = heart failure; ICD = implantable cardioverter defibrillator; IQR = interquartile range; LVEF = left ventricular ejection fraction; MI = myocardial infarction; PCI = percutaneous coronary intervention; ROSC = return of spontaneous circulation; SMD = standard mean deviation.

#### Comparison of patients with HF according to LVEF

Survival at 30 days was 35.2% for LVEF < 40%, 30.4% for LVEF 40–49%, 23.1% for LVEF ≥ 50% and 16.3% for those without a recent LVEF measurement; *p* < 0.001 (Table 2).

### Adjusted odds ratios for 30-day survival

#### Comparison of patients with HF, acute MI or both

**Figure 2:**
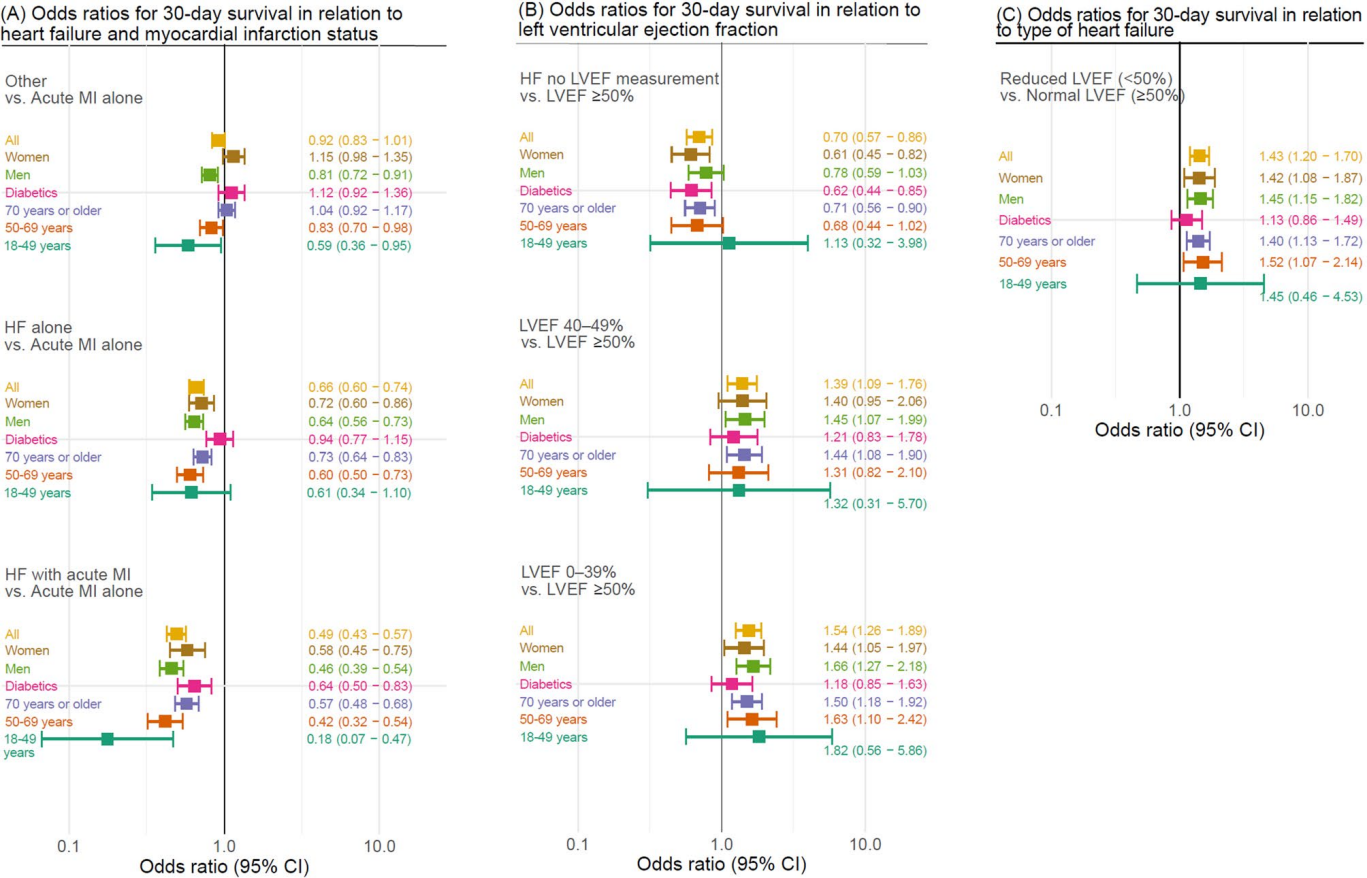
Adjusted odds ratios for 30-day survival. Odds ratios for 30-days survival, with covariate adjustment for age, sex, time from arrest to cardiopulmonary resuscitation (CPR), initial rhythm and witnessed status. Subgroup analyses were performed in relation to sex, age-group (18–49 years, 50–69 years and 70 years or older) and diabetes status. HF = heart failure; LVEF = left ventricular ejection fraction; MI = myocardial infarction.

Odds ratio (OR) for overall 30-day survival was 0.66 (95% confidence interval [CI] 0.60–0.74) for HF alone compared with acute MI alone, and 0.49 (95% CI 0.43–0.57) for HF with acute MI as compared to acute MI alone (Fig. 2A). In the subgroup of patients with diabetes, those with HF alone had an OR for 30-day survival of 0.94 (95% CI 0.77–1.15), compared with patients with diabetes with acute MI alone; for patients with HF and acute MI, the OR was 0.64 (95% CI 0.50–0.83) (Fig. 2A). For other, overall OR for 30-day survival was 0.93 (95% CI 0.83–1.01) (Fig. 2A).

#### Comparison of patients with HF alone according to LVEF

Overall, the adjusted OR for 30 day survival was 1.54 (95% CI 1.26–1.89) for LVEF < 40%, 1.39 (95% CI 1.09–1.76) for LVEF 40–49% and 0.70 (95% CI 0.57–0.86) for no recent LVEF measurement, when compared to patients with LVEF ≥ 50% (Fig. 2B). Adjusted OR for overall 30 day survival was 1.43 (95% CI 1.20–1.70) for reduced LVEF compared with normal LVEF (Fig. 2C).

**Figure 3:**
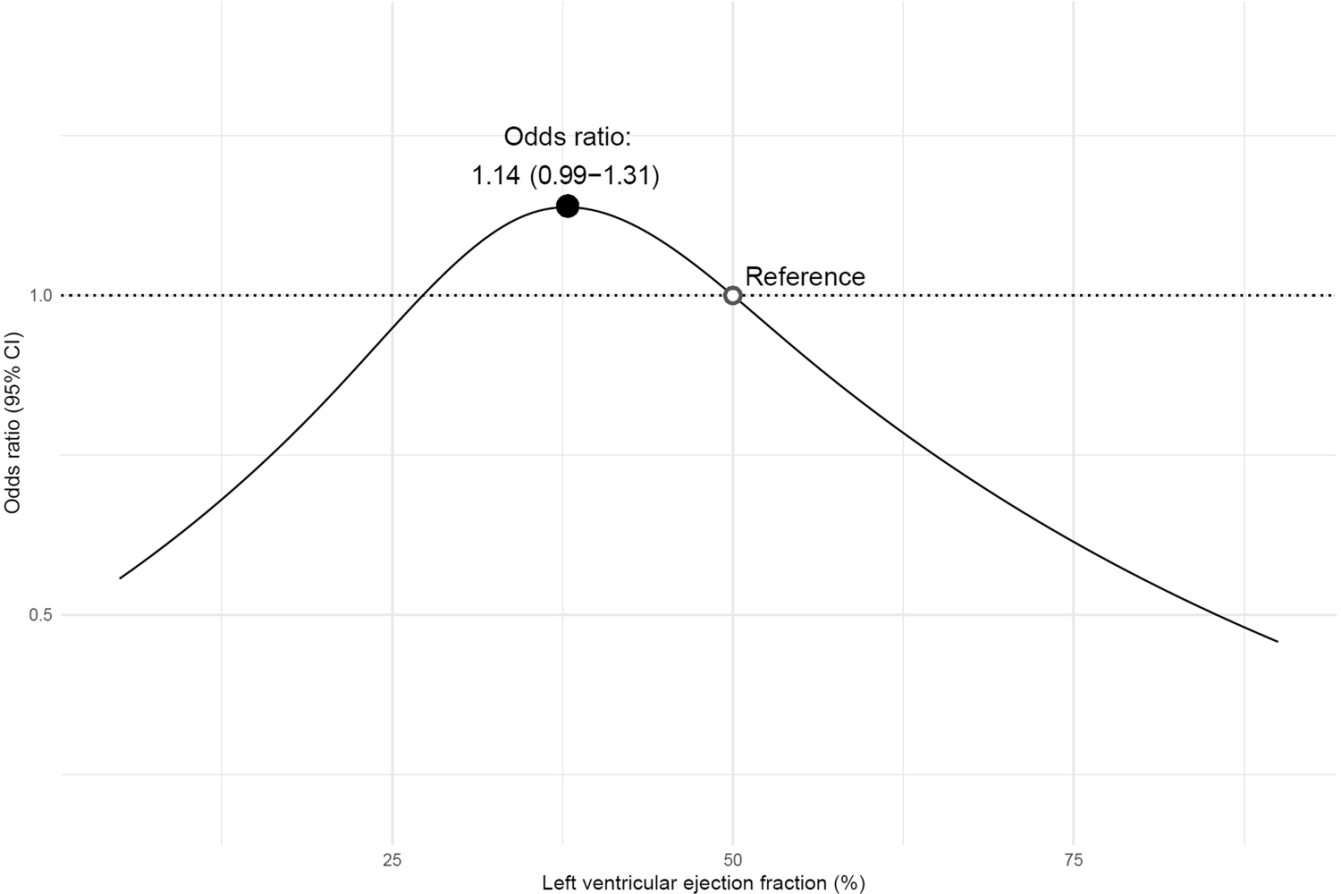
Association between LVEF and 30-days survival in patients with HF alone. Modelling the association between LVEF and 30-days survival using logistic regression. LVEF was expanded into a restricted cubic spline with 4 knots, and covariate adjustment was made for age, sex, time from arrest to start of cardiopulmonary resuscitation (CPR), initial rhythm and witnessed status. CI = confidence interval; LVEF = left ventricular ejection fraction; OR = odds ratio.

The association between LVEF and 30-day survival among patients with HF alone is shown in more detail in Fig. 3. Using patients with a LVEF of 50% as the reference group, the highest probability of survival was found in patients with a LVEF of 38% (OR 1.14 [95% CI 0.99–1.31]). As compared with the reference group, people with a LVEF < 27% or > 50% had a lower probability of survival (Fig. 3).

### Trends in 30-day survival

#### Comparison of patients with HF, acute MI or both

**Figure 4:**
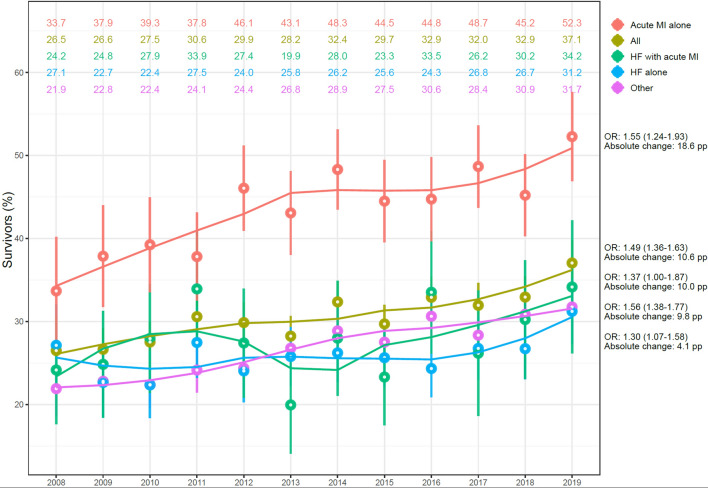
Trends in survival. Trends in 30-days survival during 2008 to 2019, in relation to HF and acute MI status. Adjustment was made for age and sex. Odds ratios (OR) were calculated by comparing cases during 2017–2019 with 2008–2010. The absolute change equals the difference between year 2008 and year 2019. HF = heart failure; MI = myocardial infarction.

From 2008 to 2019, all groups showed an increase in 30-day survival (Fig. 4). Overall, survival increased from 26.5 to 37.1%, corresponding to an odds ratio of 1.40 (95% CI 1.23–1.60) when comparing 2017–2019 with 2008–2010. The increase was most pronounced for patients with acute MI alone, who experienced a 18.4% absolute increase in survival rates, corresponding to an odds ratio of 1.55 (95% CI 1.24–1.94) for 2017–2019 vs 2008–2010. The smallest improvement was found in patients with HF alone.

### Long-term survival

#### Comparison of patients with HF, acute MI or both

**Figure 5:**
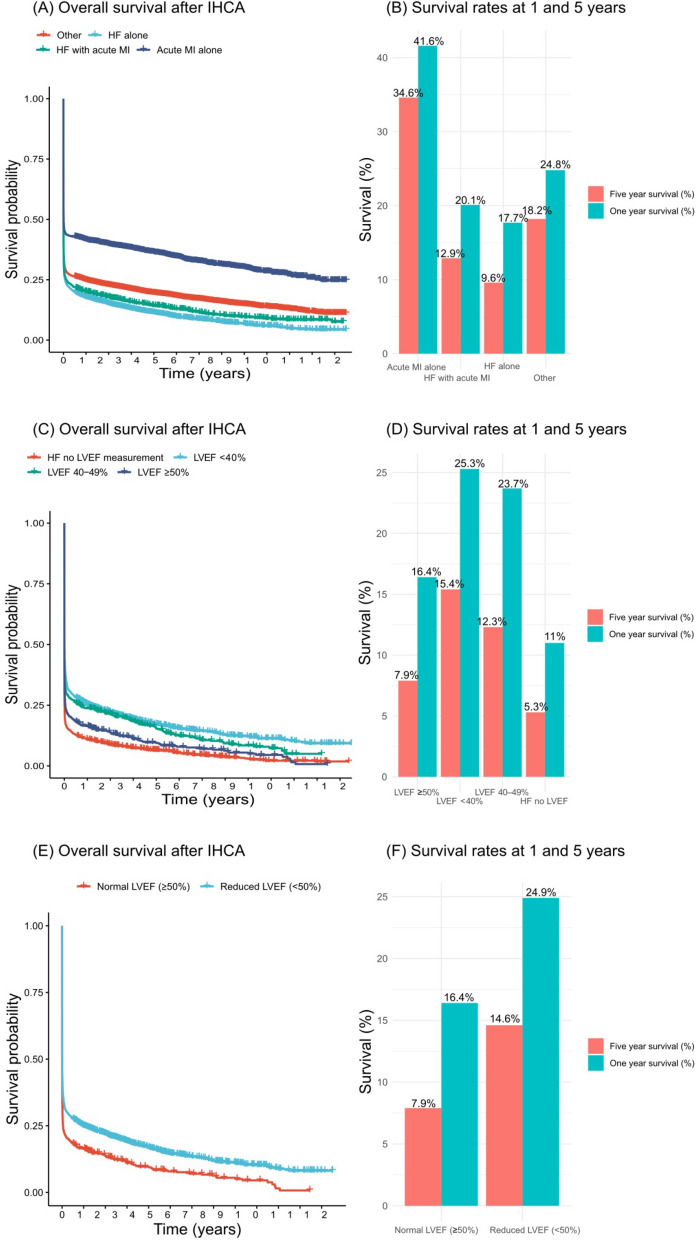
Long-term survival in relation to heart failure and acute MI status and LVEF. Kaplan–Meier estimates stratified by heart failure and acute MI status, as well as LVEF category. All figures are unadjusted. IHCA = in-hospital cardiac arrest; LVEF = left ventricular ejection fraction; MI = myocardial infarction.

The 1-year survival rate was 17.7% for HF alone, 20.1% for HF with acute MI, 41.6% for patients with an acute MI alone and 24.8% for other patients; *p* < 0.001. The 5-year survival rates were 9.6%, 12.9%, 34.6% and 18.2%, respectively for these groups; *p* < 0.001 (Fig. 5A, B). More details on survival for groups based on previous diagnosis stratified upon monitoring in Supplementary Fig. 2A-B. More details on survival for groups based on previous diagnosis stratified upon initial rhythm in Supplementary Fig. 3A-B.

#### Comparison of patients with HF alone according to LVEF

The 1-year survival rates were 25.3% for LVEF < 40%, 23.7% for LVEF 40–49%, 16.4% for LVEF ≥ 50% and 11.0% for patients without a recent LVEF-measurement; *p* < 0.001. The 5-year survival rates were 15.4%, 12.3%, 7.9% and 5.3%, respectively for these groups; *p* < 0.001 (Fig. 5C, D). Survival rates on LVEF more or less than 50% is included in Fig. 5E, F.

Survival rates for patients with an initial shockable rhythm is markedly higher among all groups compared to those without an initial shockable rhythm. For patients with an LVEF < 40% and with an initial shockable rhythm, survival at 1 year is at 44.5%, and at 5 years at 28.3%. For patients with an LVEF ≥ 50% and an initial shockable rhythm, survival at 1 year is at 36.7%, and at 5 years at 27.1%. Patients with an LVEF of 40–49% and a shockable initial rhythm had a 1 year survival rate at 48.6% and 5 years at 25.5%. The latter category also had the highest rates of survival among patients without a shockable initial rhythm (Supplementary Fig. 2D). More details on survival for groups based on LVEF stratified upon monitoring in Supplementary Fig. 2C-F. More details on survival for groups based on LVEF stratified upon initial rhythm in Supplementary Fig. 3C-F.

## Discussion

The main finding of this study was that patients with HF suffering IHCA display very poor outcomes, with only one out of ten patients surviving to five years, as compared with one out of three patients with acute MI who suffer an IHCA. Moreover, while overall survival in the 30 days following IHCA has improved during 2008 to 2019 in all groups examined, the increases in patients with HF and acute MI (41% increase) and HF alone (15% increase) were less pronounced than in individuals with acute MI alone (55% increase). We also found that patients with HFrEF, overall, had a better survival than patients with HFpEF, although there was a U-shaped relationship between LVEF and mortality, which was highest in individuals with a very low LVEF and in those with a LVEF ≥ 50%. We also found that roughly 93% of survivors among HF patients had a good neurological outcome at hospital discharge. While short-term survival differs between groups, long-term survival appears to be driven by these differences in initial success (Fig. 5). It can be argued that patients with reduced LVEF are more likely to have coronary artery disease, history of MI and myocardial scar, neurohumoral activation and electrolyte abnormalities. All these factors increase the risk of ventricular arrhythmias and, consequently, a higher prevalence of shockable initial rhythm (as we observed) and probability of survival.

Conversely, the poorer survival in patients with HFpEF could be due to location of IHCA and promptness of early management, patient factors or both. We adjusted for initial rhythm, time to CPR and witnessed status, making procedural issues less likely to explain our findings. We also adjusted for age and sex, but we did not adjust for other comorbidities. Our findings might also seem to indicate a paradox whereby patients with HFpEF, overall, have a lower mortality rate than patients with HFrEF but, when they experience an IHCA, have a lower chance of survival. However, these two observations are not incompatible as patients hospitalized with HF represent the more severe end of the clinical spectrum and, although cardiac arrest may be less common, overall in patients with HFpEF, compared with HFrEF, the causes of arrest in HFpEF may be less amenable to correction (as exemplified by the twofold difference in the rate of shockable rhythm), the patients overall may be less likely to recover or both. It can be argued that the higher survival rate among patients with HFrEF compared to HFpEF is due to the differences in initial rhythm, and though this is a factor, our sensitivity analysis stratified upon initial rhythm would suggest this not the entire case behind the differences in survival between different HF phenotypes, since patients with HFpEF and an initial shockable rhythm has a moderately lower survival rate at 1 year compared to patients with HFrEF and an initial shockable rhythm (Supplementary Fig. 3D).

Patients with a very low LVEF also displayed high mortality. This may also reflect a different type of initial electrical event or just the known poor prognosis of patients with severe left ventricular systolic dysfunction and the difficulty in such sick heart recovering contractile function after a period of arrest^3^.

Levy and colleagues studied factors associated with good neurological outcome at discharge for patients with acute HF and IHCA during the period 2000 to 2007. They reported that 20.0% survived to hospital discharge, and among those, 88.5% had good neurological outcome^23^. Our data from 2008–2009 shows 30-day survival around 20–25%, and this percentage has increased, in absolute numbers, by almost 1% annually (Fig. 4). With regards to neurological function, we show that 92.0% and 93.3% of patients with HF alone and HF with acute MI, respectively, have a good neurological outcome at discharge. These are encouraging findings since they highlight that successful resuscitation attempts are very likely to result in good neurological outcome. Also, previous studies have shown that while patients with HF fear the potential neurological disabilities, the majority would choose to have cardiopulmonary resuscitation should they need it^24,25^.

This study is limited due to being a retrospective observational study, and thus affected by the limitations of such design. The SRCR lacks data on medication, and only few comorbidities are recorded. Moreover, data on LVEF was only available for 55% of cases with HF alone. It is possible that these cases represent a selected subgroup. This is suggested by the fact that cases with no recent LVEF measurement displayed different clinical characteristics (including being older) and worse survival. HF patients in Sweden typically undergo echocardiographic examination once yearly, which implies that some LVEF measurement are randomly missing, while others will have missing measurements due to non-random reasons; the group with missing measurements was presumably enriched with patients with less active care, in more advanced stages of heart failure.

## Conclusion

To conclude, we show that although survival is very low in heart failure patients who suffer an IHCA, with patients with low and high LVEF at highest risk of death, survival is improving over time and neurological function among survivors is good.

## Supplementary Information

Supplementary Information.
