# Supplementary material for: Clinical characteristics and survival in patients with heart failure experiencing in hospital cardiac arrest
Source: Sci Rep. 2022 Apr 5;12:5685. doi: 10.1038/s41598-022-09510-4 (PMC8983650; doi:10.1038/s41598-022-09510-4)
Supplement: Supplementary file 1 — Supplementary Information. [file 41598_2022_9510_MOESM1_ESM.docx]

**Clinical Characteristics and Survival in Patients with Heart Failure Experiencing In Hospital Cardiac Arrest**

Emma Aune^1*^, John McMurray^2^, Peter Lundgren^1,3^, Naveed Sattar^2^, Johan Israelsson^4,5^, Per Nordberg^6^, Johan Herlitz^3,7^, Araz Rawshani^1,7^

^1^ Institute of Medicine, University of Gothenburg, Gothenburg, Sweden

^2^ British Heart Foundation (BHF) Cardiovascular Research Centre, University of Glasgow, Glasgow, United Kingdom

^3^ Prehospen- Centre for Prehospital Research, University of Borås, Borås, Sweden

^4^ Department of Internal Medicine, Division of Cardiology, Kalmar County Hospital, Region Kalmar County, Sweden

^5^ Faculty of Health and Life Sciences, Linnaeus University, Kalmar, Sweden

^6^ Department of Medicine, Center for Resuscitation Science, Karolinska Institute, Solna, Sweden

^7^ The Swedish Registry of Cardiopulmonary Resuscitation, Gothenburg, Sweden

***Corresponding author**

Emma Aune, emma.aune@gu.se
Department of Molecular and Clinical Medicine
Institute of Medicine
University of Gothenburg

# Supplementary material


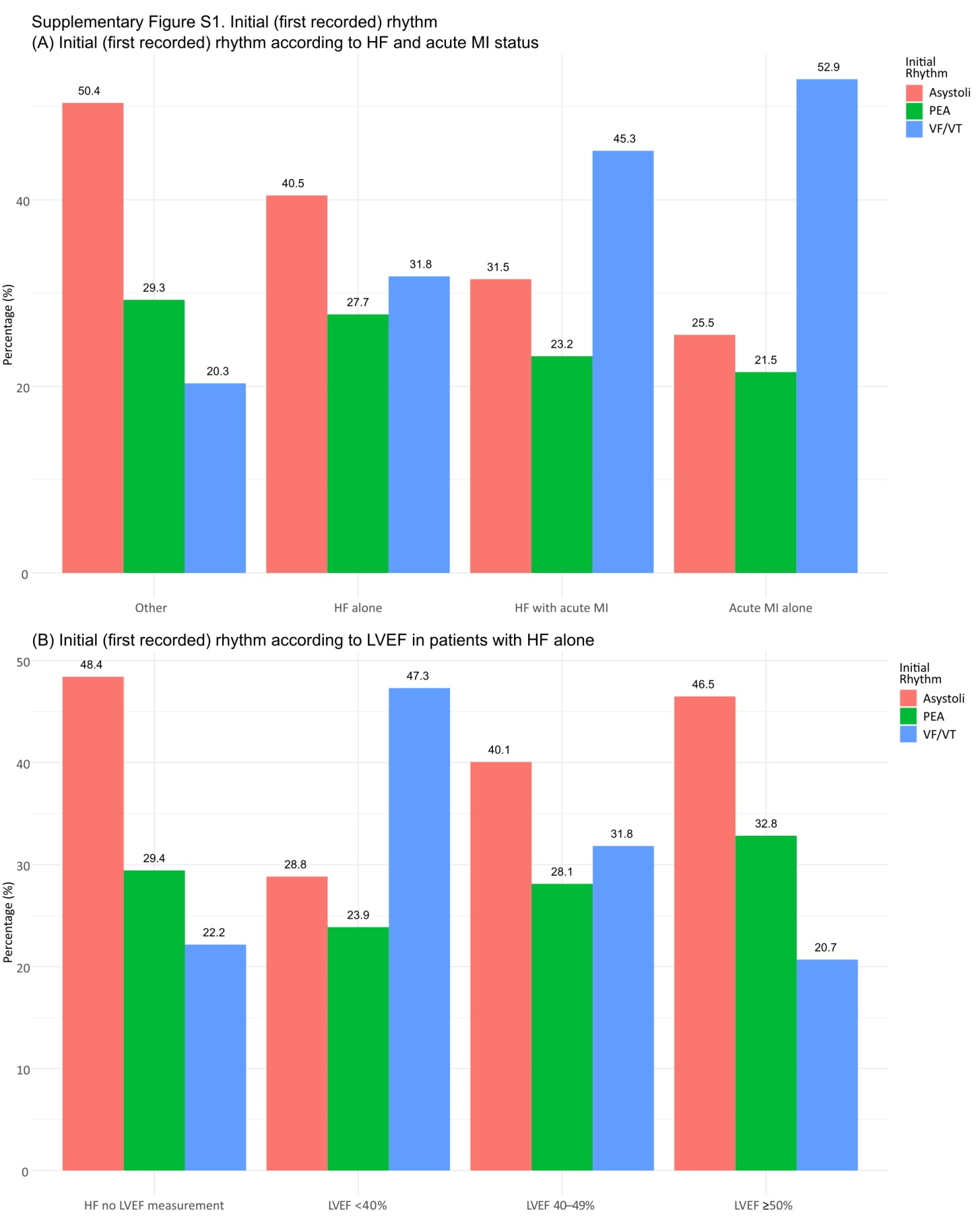


Supplementary material, Figure S1. **Initial rhythm in relation to heart failure and acute myocardial infarction status or in patients with heart failure alone, stratified by left ventricular ejection fraction (LVEF)**

Initial (first recorded) rhythm according to the first ECG recording, and ignoring the information produced by the connected defibrillator. Proportions of shockable (VF/VT) and non-shockable (PEA, asystole) rhythms differs from percentages in Table 1, since the latter also accounts for information produced by the defibrillator.

*Abbreviations*: ECG = electrocardiogram; HF = heart failure; MI = myocardial infarction; PEA = pulseless electrical activity; VF = ventricular fibrillation; VT = ventricular tachycardia.


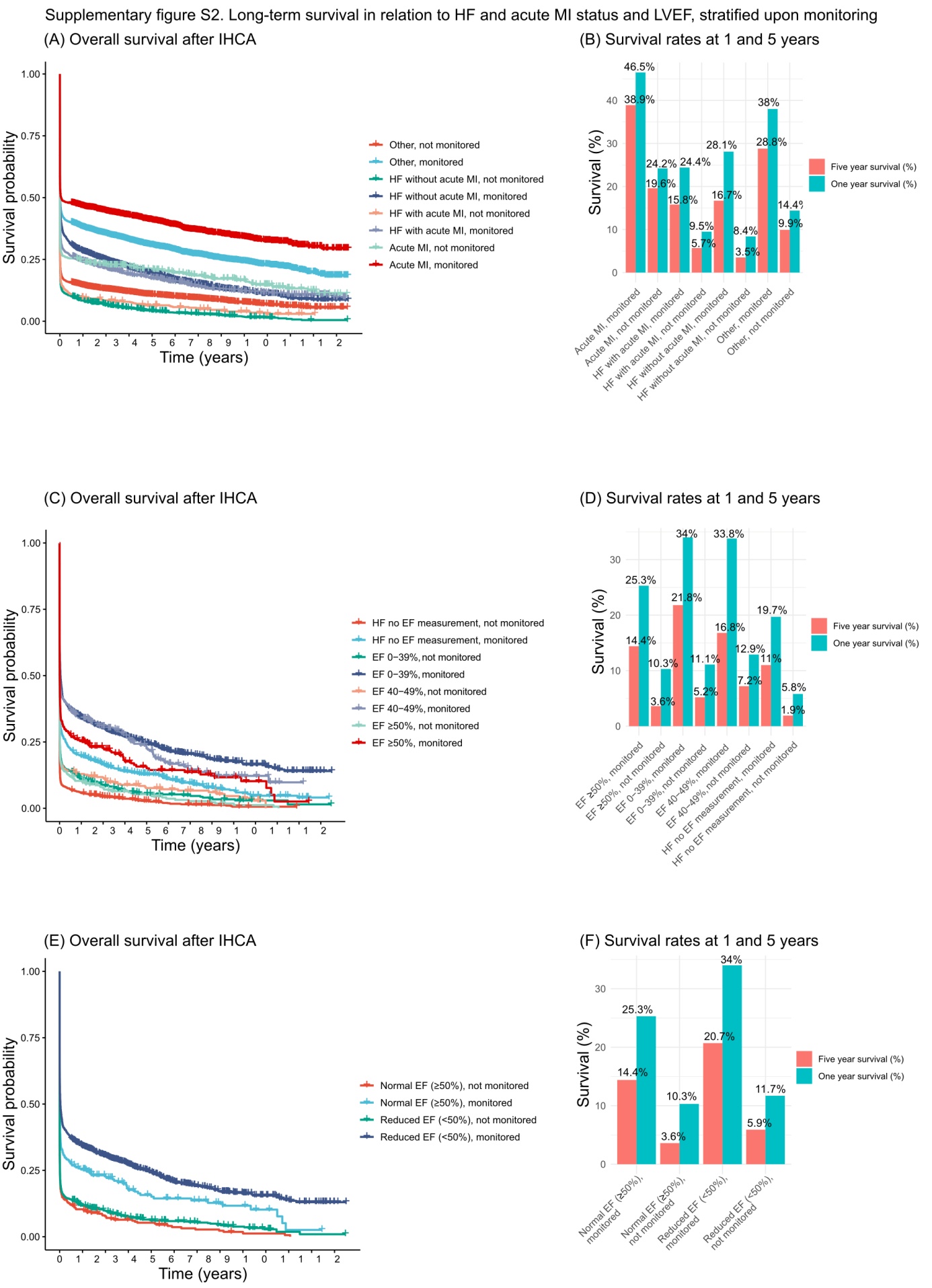


Supplementary material, Figure S2. **Long-term survival in relation to heart failure and LVEF stratified upon monitoring at time of IHCA**

Kaplan-Meier estimates stratified by heart failure and acute MI status, as well as LVEF category, and by monitoring status at time of IHCA. All figures are unadjusted.

*Abbreviations*: IHCA = in-hospital cardiac arrest; LVEF = left ventricular ejection fraction; MI = myocardial infarction.


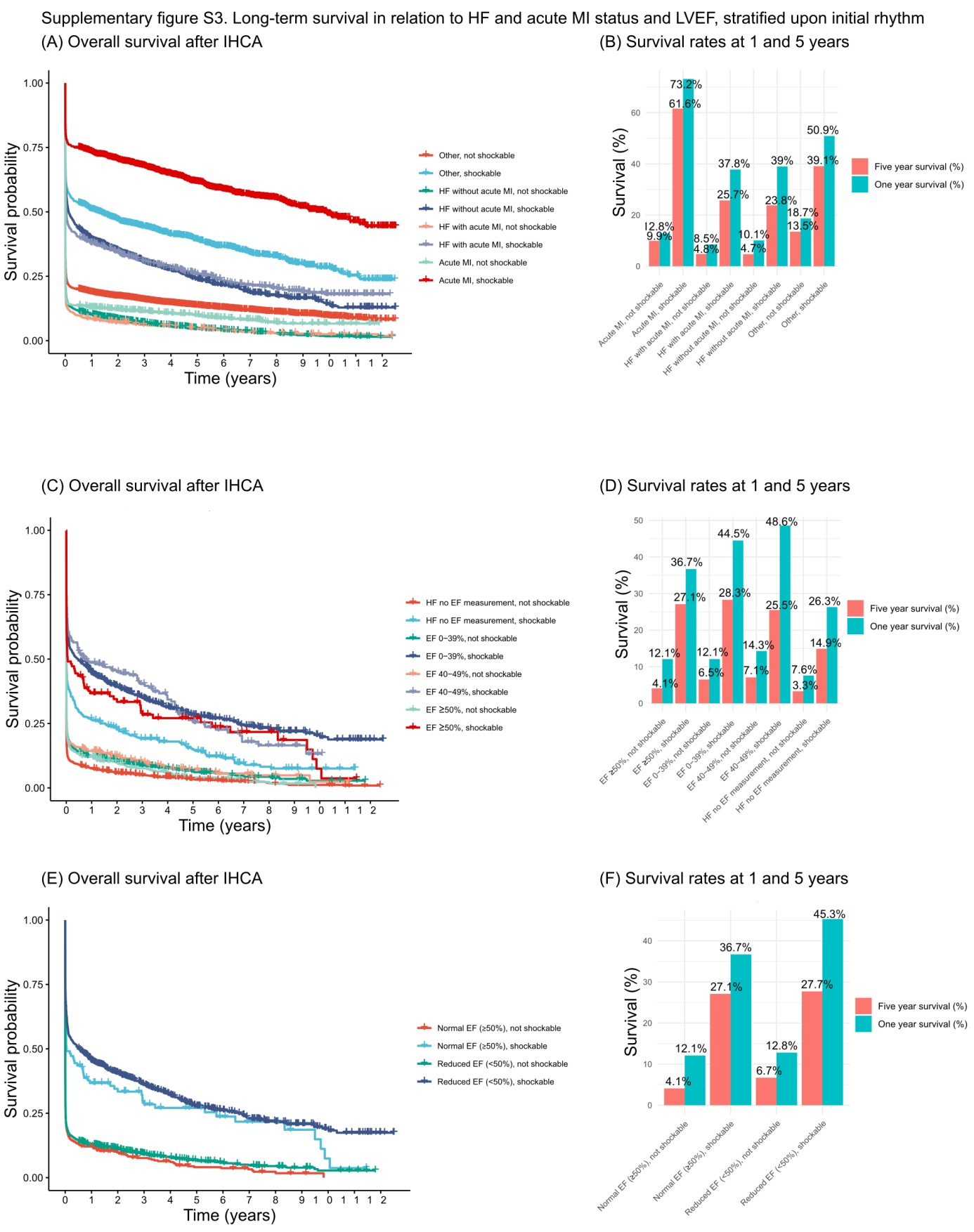


Supplementary material, Figure S3. **Long-term survival in relation to heart failure and LVEF stratified upon initital rhythm during IHCA**

Kaplan-Meier estimates stratified by heart failure and acute MI status, as well as LVEF category, and by shockble or non-shockable initial rhythm during IHCA. All figures are unadjusted.

*Abbreviations*: IHCA = in-hospital cardiac arrest; LVEF = left ventricular ejection fraction; MI = myocardial infarction.
